# Effect of Spherical Aberration on the Optical Quality after Implantation of Two Different Aspherical Intraocular Lenses

**DOI:** 10.1155/2017/8039719

**Published:** 2017-08-16

**Authors:** Michael Lasta, Kata Miháltz, Illés Kovács, Pia Veronika Vécsei-Marlovits

**Affiliations:** ^1^Department of Ophthalmology, Hospital Hietzing, Vienna, Austria; ^2^Karl Landsteiner Institute of Process Optimization and QM in Cataract Surgery, Vienna, Austria; ^3^Department of Ophthalmology, Semmelweis University, Budapest, Hungary

## Abstract

**Purpose:**

To compare the effect of spherical aberration on optical quality in eyes with two different aspherical intraocular lenses.

**Methods:**

120 eyes of 60 patients underwent phacoemulsification. In patients' eyes, an aberration-free IOL (Aspira-aA; Human Optics) or an aberration-correcting aspherical IOL (Tecnis ZCB00; Abott Medical Optics) was randomly implanted. After surgery, contrast sensitivity and wavefront measurements as well as tilt and decentration measurements were performed.

**Results:**

Contrast sensitivity was significantly higher in eyes with Aspira lens under mesopic conditions with 12 cycles per degree (CPD) and under photopic conditions with 18 CPD (*p* = 0.02). Wavefront measurements showed a higher total spherical aberration with a minimal pupil size of 4 mm in the Aspira group (0.05 ± 0.03) than in the Tecnis group (0.03 ± 0.02) (*p* = 0.001). Strehl ratio was higher in eyes with Tecnis (0.28 ± 0.17) with a minimal pupil size larger than 5 mm than that with Aspira (0.16 ± 0.14) (*p* = 0.04). In pupils with a minimum diameter of 4 mm spherical aberration had a significant effect on Strehl ratio, but not in pupils with a diameter less than 4 mm.

**Conclusions:**

Optical quality was better in eyes with the aberration-correcting Tecnis IOL when pupils were large. In contrast, this could not be shown in eyes with pupils under 4 mm or larger. This trial is registered with Clinicaltrials.gov NCT03224728.

## 1. Introduction

In recent years, cataract surgery has become a highly precise and safe surgical procedure due to phacoemulsification technique and advanced intraocular lens (IOL) designs. To face increasing demands on visual and refractive outcome, efforts have been made to further improve IOL designs. Today, it is widely recognized that implantation of spherical IOLs leads to increased spherical aberrations (SA), therefore decreasing retinal image quality [1]. Aspherical IOLs have been developed in order to overcome this issue. Aberration-correcting aspherical IOLs are aiming to compensate for the corneal spherical aberration of the eye. It is known that aspherical lenses significantly decrease higher-order aberrations (HOA) postoperatively [1]. A number of studies have shown that aspherical IOLs perform better compared to conventional spherical ones in terms of contrast sensitivity (CS), particularly under mesopic conditions [2, 3]. Furthermore it is known that a precise position of an aspherical IOL is essential for perfect optical quality [4]. IOL displacement such as tilt and decentration could not only impair its aberration-correcting effect but may even induce additional optical aberrations [4, 5].

The question whether correction of the total SA of the eye or some residual SA leads to better visual outcome and better optical quality remains to be elucidated. It is known that healthy young eyes with good visual acuity have some positive ocular SA [6]. However, results of other studies indicate that a postoperative SA of zero should be aimed in order to optimize visual outcome [3, 7].

Aberration-free aspherical IOLs follow another concept of aspherical IOL design. These implants have no inherent SA; thus, they do not introduce additional SA into the optical system of the eye. Some studies indicate that aberration-free aspheric IOLs may result in better optical quality compared to aberration-correcting aspheric IOLs—even in case of decentration and tilt of the IOL [8]. Our study aimed to evaluate the optical quality and potential benefits on visual outcome of an aberration-free aspherical IOL compared to those of an aspherical IOL with negative SA.

Optical quality is a subjective construct that can only be described indirectly by objective metrics such as wavefront error measurements and visual quality metrics or functional data like visual acuity and contrast sensitivity. Wavefront analysis isolates the effect of lower-order aberrations (defocus and astigmatism) and higher-order aberrations as well as the contribution of individual aberrations on optical quality. Modulation transfer function (MTF) and Strehl ratio are well related to the image quality of an optical system, including the human eye [9].

In this study, an aberration-free aspherical IOL (Aspira-aA, Human Optics AG; Erlangen, Germany) was compared to an aberration-correcting aspherical IOL with negative SA (Tecnis ZCB00, Abbott Medical Optics; Illinois, USA) regarding the effect of spherical aberration on visual outcome and optical quality.

## 2. Methods and Subjects

This prospective single-center study was conducted at the Department of Ophthalmology, Hospital Hietzing, Vienna, Austria, in accordance with the Declaration of Helsinki. The study received approval from the Ethics Committee of the City of Vienna, and each subject gave written informed consent before taking part.

Before inclusion, all patients underwent a full ophthalmic assessment including slit lamp examination and funduscopy. Intraocular eye pressure was measured with the Goldmann applanation tonometry. Optical biometry was performed using the IOLMaster 500 (Carl Zeiss Meditec AG; Jena, Germany) for axial length and corneal radii of curvature measurements. For IOL calculation, SRK®/T, Holladay I, and Hoffer® Q were used, depending on the length of the patients' eyes.

Patients with bilateral age-related cataract and an age range between 50 and 80 years were included. Eyes with relevant other ocular pathologies or previous surgeries, a potential postoperative corrected distance visual acuity worse than 20/20, and corneal astigmatism of 1.5 diopters and more were excluded from the study. For patients' subjective refraction, a plus cylinder design was used. To be able to compare IOL function, patients had to manifest similar corneal asphericity.

37 female and 23 male subjects were enrolled in this trial. They were randomly assigned to implantation of the aberration-free aspherical Aspira-aA in one eye and the aberration-correcting aspherical Tecnis ZCB00 in the fellow eye. Both IOLs are monofocal acrylic one-piece lenses with C-loop haptic design.

The eye with the worse visual acuity was operated first, followed by the second eye one week afterwards. All of our patients have been corrected for emmetropy after surgery.

### 2.1. Surgical Technique

Surgery was performed in topical anesthesia using oxybuprocaine eye drops. The self-sealing 2.4 mm incision, capsulorrhexis, phacoemulsification, irrigation and aspiration of cortical material, and injection of viscoelastic substance into the capsular bag were performed as standard procedures. The IOL was implanted via an injector into the capsular bag followed by thorough aspiration of the viscoelastic substance from the eye.

### 2.2. Postoperative Assessments

Three months postoperatively, the following measurements were carried out in each subject:

Uncorrected distance visual acuity (UDVA) and corrected distance visual acuity (CDVA) were measured using the ETDRS chart at a viewing distance of four meters. In addition, monocular defocus curve measurements were performed with the best distance correction adding glasses in 0.50-diopter increments from +0.5 D to −3.00 diopters.

Photopic and mesopic contrast sensitivity was evaluated with best refraction using the Optec 6500 contrast sensitivity tester (Stereo Optical Co.; Chicago, Illinois, USA) under photopic (85 cd/m^2^) and mesopic (3 cd/m^2^) conditions. This item uses functional acuity contrast charts (FACT) testing five spatial frequencies and nine levels of contrast. The patient determines the last grating seen for each row (A, B, C, D, and E) and reports the orientation of the grating: right, up, or left. The last correct grating seen for each spatial frequency is plotted on a contrast sensitivity curve.

Thereafter, pupils were dilated using one eye drop containing 0.5% tropicamide and one eye drop containing 10% phenylephrine.

To assess optical quality characteristics including higher-order aberrations, wavefront measurements were obtained using the HOYA iTrace™ Surgical Workstation (HOYA Surgical Optics GmbH; Frankfurt, Germany).

The HOYA iTrace Surgical Workstation provides an objective clinical evaluation of the eye's optical quality. Performing these measurements with maximum-dilated pupils, we calculated all relevant parameters with a pupil size of two, three, four, and five millimeters.

The following parameters have been calculated:

Wavefront aberrometry describes the optical properties of the eye in individual Zernike polynomials. Optical quality can be described by metrics of image quality for point objects (point spread function (PSF)) and for grating objects (modulation transfer function (MTF)). The iTrace is able to measure the following image quality metrics: PSF, Strehl ratio, and MTF.

Strehl ratio is the ratio of the peak height of the PSF divided by the maximum intensity of PSF in the diffraction-limited perfect eye. Strehl ratio ranges from 0 to 1; the greater Strehl ratio, the better the quality of vision [10]. By the iTrace, Strehl ratio is calculated from the retinal PSF. The MTF is the modulus of the Fourier transform of the PSF. It characterizes the degradation of the image for every spatial frequency of the object.

Root mean square (RMS) error was calculated by the iTrace software from Zernike coefficients. The total HO (higher order) is the RMS of higher-order terms (Z3 to Z6).

IOL tilt and decentration were measured with the Visante anterior segment OCT (Carl Zeiss Meditec AG; Jena, Germany). Images were obtained in mesopic conditions for each examination. Anterior single-scan mode was used to assess pictures. These were obtained in four axes (180 to 0 degrees, 45 to 225 degrees, 315 to 135 degrees, and 270 to 90 degrees). Tilt and decentration of IOLs were calculated accordingly as previously described by Rosales et al. [11].

### 2.3. Statistical Analysis

Statistical analyses were performed with Statistica 12.0 (Statsoft Inc.; Tulsa, OK, USA). The Shapiro-Wilk *W* test was used to confirm normal distribution of the variables. Paired-samples *t*-test was used to compare means between the eyes of the same subject. This test allows for comparing within-subject parameters (visual acuity, keratometric, OCT, and intraocular optical quality parameters) in the two study groups by taking into account between-eye correlations by treating data from the two eyes of the same patient in statistical analyses as repeated measures.

To determine the effect of intraocular spherical aberration on visual quality in relation to the pupillary diameter, multivariable regression analysis using the generalized estimating equation (GEE) model was performed where data from patients who had measurements in both eyes were statistically analyzed as repeated measures. The type of the IOL and corneal spherical aberration were included as confounders in these multivariable regression models to adjust for their effect on total ocular Strehl ratio. Model fit was assessed using the value of the corrected quasilikelihood under independence model criterion (QICC) with a lower QICC values indicating a better fit to data. In all analyses, a *p* value less than 0.05 was considered statistically significant.

## 3. Results

The age range of the included patients was between 47 and 79 years (mean 69 ± 7.8 years). For the three-month follow-up, data of 47 persons were eligible for evaluation.

Preoperative findings of the patients are shown in Table 1.

Three months after phacoemulsification, mean corrected distance visual acuity (CDVA) increased in all patients (logMAR 0.073 ± 0.092 in eyes with Aspira and 0.040 ± 0.098 in eyes with Tecnis. There was no significant difference between both IOL groups (*p* = 0.22).

In all patients, postoperative refraction showed a slight myopic shift. The difference between planned and manifest postoperative sphere was found in a majority of the eyes; however, we could not find a significant difference between both lenses.

In addition, we measured no significant difference in depth of focus between Aspira and Tecnis IOLs.

Postoperative characteristics are shown in Table 2.

### 3.1. Contrast Sensitivity

In eyes with the aberration-free Aspira, higher contrast sensitivity under photopic as well as under mesopic conditions was measured compared to that in eyes with Tecnis IOL. However, only one out of 5 measurements under photopic conditions with 18 cycles per degree (CPD) as well as one measurement under mesopic conditions with 12 CPD presented a significant difference between both IOLs (Table 3).

### 3.2. Tilt and Decentration

Three months after implantation, both IOLs showed a slight tendency to decentration in temporal direction; however, no significant difference between lenses was found (Table 4).

Vertical tilt was higher in Aspira than in Tecnis IOL. However, this finding did not reach the level of significance. In contrast, horizontal tilt was significantly lower in Aspira compared to Tecnis (0.35 ± 1.65° and 0.67 ± 1.42°, resp.; *p* = 0.02; Table 4).

### 3.3. Wavefront Measurements

All wavefront measurements were performed with a pupil diameter of 5 mm. Data with a pupil size of 2, 3, and 4 mm were calculated with the software program.

Higher-order root mean square (HORMS) showed no significant difference between the two lenses. From 0.054 ± 0.042 in Aspira and 0.050 ± 0.036 in Tecnis with a pupil size of 2 mm, values increased to 0.52 ± 0.25 and 0.56 ± 0.52 with a maximum-dilated pupil.

In contrast, total spherical aberration (TSA) was significantly higher in Aspira compared to Tecnis. However, this was only shown with pupil sizes of minimum 4 mm (*p* = 0.001; Table 5).

Total Strehl ratio was comparable in both lenses with a pupil size of 2, 3, and 4 mm. However, with a pupil size of 5 mm, we calculated significantly higher values in eyes with Tecnis IOL than in eyes with Aspira IOL (0.16 ± 0.14 versus 0.08 ± 0.17, resp.; *p* = 0.04). This effect was shown in modulation transfer function as well (0.46 ± 0.13 versus 0.39 ± 0.15; *p* = 0.04). However, these differences did not reach the level of significance after a Bonferroni correction.

Multivariate regression models showed that total spherical aberration had a significant negative effect (beta: −1.54, 95% CL: −2.56 to −0.51; *p* = 0.004) on total ocular Strehl ratio when the pupillary diameter was ≥4 mm, but not when pupillary diameter was less than 4 mm after adjustment for the effect of IOL type and corneal spherical aberration.

## 4. Discussion

The surgical technique of phacoemulsification has been improved continuously. The measure of successful surgery is more than just an increased visual quality. Patients expect high optical quality without disturbing aberrations as well as high contrast sensitivity during night and day.

A various number of spherical and aspherical IOL designs have been developed aiming to achieve perfect optical quality.

In this trial we compared the aberration-free aspherical Aspira-aA with the aberration-correcting aspherical Tecnis ZCB00.

Several authors mentioned that there is increased optical quality and subjective satisfaction in patients with aspherical lenses compared to those with spherical ones [12, 13]. In the clinical trial of Santhiago et al. [12], the aspherical aberration-free Akreos AO was compared with spherical Akreos Fit. The authors described significantly higher contrast sensitivity and decreased aberrations in the aspherical lens.

Apart from comparisons between spherical and aspherical IOLs, a few authors compared different aspherical lenses with each other.

In the work of Rajabi et al. [14], the Aspira-aA was compared with Akreos AO. In contrast to our findings, they mentioned that there was no significant difference in higher-order aberrations between the two lenses. Due to the fact that they compared two IOLs with similar SA, the major question (what is the ideal SA regarding optical quality) could not be answered.

Johansson et al. [15] evaluated Akreos AO and Tecnis Z9000. Although the Tecnis showed less aberration than the Akreos, more patients reported better subjective quality with Akreos IOL. The same IOLs were evaluated in the work of Baghi et al. [16]. In this trial, Akreos AO causes significantly more spherical aberration in pupil diameter of 4 and 6 mm. This seems to be in accordance with our findings, where Aspira lens causes higher spherical aberrations in pupil diameters of 4 and 5 mm.

Nochez and coworkers [17] stated that an IOL with a spherical aberration of zero leads to better optical quality and even better MTF contrast than aberration-correcting intraocular lens. However, the best compromise of subjective depth of contrast and objective contrast sensitivity is reached with a total spherical aberration between 0.07 *μ*m and 0.10 *μ*m. We found a total spherical aberration between 0.006 *μ*m with a pupil size of 2 mm and 0.11 *μ*m with a pupil size of 5 mm.

In general a higher spherical aberration decreases Strehl ratio resulting in lower optical quality [10]. We can reassure this phenomenon as our multivariable regression models showed that intraocular spherical aberration had significant negative effect on total ocular Strehl ratio. Nevertheless, it has to be mentioned that this effect applied only when the pupillary diameter was 4 mm and more. With pupil sizes of less than 4 mm, adjustment for the effect of IOL type and corneal spherical aberration did not show this effect. We can assume that spherical aberration influences Strehl ratio and optical quality less when pupil sizes are small due to the fact that small pupils present smaller values of spherical aberration. In contrast, higher spherical aberration significantly influences optical quality when the pupils are small.

Results of evaluating contrast sensitivity between aspherical lenses differ in various studies. Shentu et al. [18] could not find any difference in contrast sensitivity between the two aspherical lenses. Our results show significant differences in contrast sensitivity, although this was found at 18 CPD under photopic conditions and at 12 CPD under mesopic conditions. Hence, it can be assumed that patients might not notice any distinction in their daily life.

Jia and Li [19] mentioned that a presurgical measurement of corneal spherical aberration should be performed to determine which type of IOL would lead to optimal optical quality. A possible solution for this problem would be to choose the SA of the implanted IOL individually as suggested by Jia and Li.

Tilt and decentration of IOLs may lead to higher spherical aberration resulting in decreased optical quality [20]. In our study, three months postoperatively, a significantly higher horizontal tilt of Tecnis lens was measured. In contrast, both lenses did not differ in vertical tilt as well as decentration, although a horizontal temporal decentration were found in both eyes. These findings comply with the work of Rosales et al. [11]. They described that IOL decentration tends to be mirror symmetric between the right and left eye.

Study limitations are that only total aberrations of study eyes were calculated. Further, in order to obtain precise wavefront measurements, detailed knowledge about internal aberrations would be essential, which will be subject of future investigations. Nevertheless, we aimed to constitute the total efficiency of pseudophakic eyes. Furthermore, we included patients with similar corneal asphericity in order to compare both IOLs. Regarding statistics, Bonferroni calculations may have been more suitable concerning data calculations, which is considered as a drawback of the current study.

### 4.1. Conclusion

We can conclude that aberration-free as well as aberration-correcting aspherical intraocular lenses feature high optical quality conditions. When pupils are large, eyes with aberration-correcting Tecnis IOL showed higher modulation transfer function and Strehl ratio values than eyes with aberration-free Aspira lens. Nevertheless, it has to be clarified that this significance is not shown after a Bonferroni correction. Moreover, this benefit could not be verified in eyes with pupils under 4 mm.

## Figures and Tables

**Table 1:** Patients' preoperative characteristics (mean ± SD; *n* = 60).

	Aspira-aA	Tecnis ZCB00	*p* value
CDVA (logMAR)	0.38 ± 0.13	0.39 ± 0.14	0.85
Sphere (D)	−0.50 ± 2.32	−0.42 ± 2.63	0.43
Cylinder (D)	0.68 ± 0.61	0.74 ± 0.5	0.40
Axial eye length (mm)	23.40 ± 0.99	23.45 ± 1.05	0.87

CDVA: corrected distance visual acuity.

**Table 2:** Patients' postoperative characteristics (mean ± SD; *n* = 60).

	Aspira-aA	Tecnis ZCB00	*p* value
UDVA (logMAR)	0.14 ± 0.13	0.12 ± 0.15	0.25
CDVA (logMAR)	0.07 ± 0.09	0.04 ± 0.09	0.22
Sphere ∆ (D)	−0.22 ± 0.67	−0.50 ± 0.52	0.29
Depth of focus (D)	0.61 ± 0.33	0.65 ± 0.29	0.74
Corneal asphericity with a pupil size of 4 mm	0.19 ± 0.24	0.26 ± 0.16	0.19

UDVA: uncorrected distance visual acuity; CDVA: corrected distance visual acuity; sphere ∆: difference between scheduled and manifest postoperative sphere.

**Table 3:** Contrast sensitivity values in the eyes, implanted with Aspira or Tecnis IOLs. *p*: Student's *t*-test on dependent samples (log; mean ± SD; *n* = 60).

	Aspira-aA	Tecnis ZCB00	*p* value
Photopic			
CPD 1.5	1.54 ± 0.20	1.52 ± 0.17	0.62
CPD 3	1.78 ± 0.20	1.76 ± 0.18	0.60
CPD 6	1.72 ± 0.27	1.74 ± 0.20	0.77
CPD 12	1.27 ± 0.49	1.29 ± 0.39	0.85
CPD 18	0.52 ± 0.51	0.36 ± 0.41	*0.02*
Mesopic			
CPD 1.5	1.60 ± 0.22	1.60 ± 0.20	0.92
CPD 3	1.73 ± 0.23	1.69 ± 0.24	0.40
CPD 6	1.36 ± 0.64	1.49 ± 0.33	0.22
CPD 12	0.71 ± 0.61	0.48 ± 0.58	*0.02*
CPD 18	0.09 ± 0.13	0.16 ± 0.09	0.19

CDP: cycles per degree.

**Table 4:** Results of Visante OCT measurements (mean ± SD; *n* = 60).

	Aspira-aA	Tecnis ZCB00	*p* value
Horizontal tilt (°)	0.35 ± 1.65	0.67 ± 1.42	*0.02*
Vertical tilt (°)	0.63 ± 1.76	0.19 ± 1.24	0.35
Horizontal decentration (mm)	0.03 ± 0.16	0.04 ± 0.27	0.94
Vertical decentration (mm)	0.08 ± 0.15	0.07 ± 0.23	0.96

**Table 5:** Total ocular higher-order spherical aberrations measured at different pupillary diameters in eyes implanted with Aspira or with Tecnis IOL (*μ*m; mean ± SD; *n* = 60).

	Aspira-aA	Tecnis ZCB00	*p* value
2 mm	0.006 ± 0.004	0.006 ± 0.005	0.88
3 mm	0.02 ± 0.01	0.02 ± 0.01	0.9
4 mm	0.05 ± 0.03	0.03 ± 0.02	*0.001*
5 mm	0.11 ± 0.05	0.07 ± 0.05	*0.001*

## References

[1] Yagci R., Uzun F., Acer S., Hepsen I. F. (2014). Comparison of visual quality between aspheric and spherical IOLs. *European Journal of Ophthalmology*.

[2] Assaf A., Kotb A. (2010). Ocular aberrations and visual performance with an aspheric single-piece intraocular lens: contralateral comparative study. *Journal of Cataract and Refractive Surgery*.

[3] Ohtani S., Miyata K., Samejima T., Honbou M., Oshika T. (2000). Intraindividual comparison of aspherical and spherical intraocular lenses of same material and platform. *Ophthalmology*.

[4] Eppig T., Scholz K., Löffler A., Messner A., Langenbucher A. (2009). Effect of decentration and tilt on the image quality of aspheric intraocular lens designs in a model eye. *Journal of Cataract and Refractive Surgery*.

[5] Choi S. K., Kim J. H., Lee D., Park S. H., Maeda N., Ma K. J. (2010). IOL tilt and decentration. *Ophthalmology*.

[6] Thibos L. N., Hong X., Bradley A., Cheng X. (2002). Statistical variation of aberration structure and image quality in a normal population of healthy eyes. *Journal of the Optical Society of America A: Optics, Image Science, and Vision*.

[7] Tuzcu E. A., Erkilic K., Bulut B., Ilhan N. (2013). Comparing the effect of two different intraocular lenses on optical aberrations in bilaterally operated eyes for cataract. *Journal of Medical Sciences*.

[8] Pieh S., Fiala W., Malz A., Stork W. (2009). In vitro strehl ratios with spherical, aberration-free, average, and customized spherical aberration-correcting intraocular lenses. *Investigative Ophthalmology & Visual Science*.

[9] Moreno L. J., Piñero D. P., Alío J. L., Fimia A., Plaza A. B. (2009). Double-pass system analysis on the visual outcomes and optical performance of an apodized diffractive multifocal intraocular lens. *Journal of Cataract and Refractive Surgery*.

[10] Lombardo M., Lombardo G. (2010). Wave aberration of human eyes and new descriptors of image optical quality and visual performance. *Journal of Cataract and Refractive Surgery*.

[11] Rosales P., De Castro A., Jiménez-Alfaro I., Marcos S. (2010). Intraocular lens alignment from purkinje and Scheimpflug imaging. *Clinical & Experimental Optometry*.

[12] Santhiago M. R., Netto M. V., Barreto J. (2010). Wavefront analysis, contrast sensitivity, and depth of focus after cataract surgery with aspherical intraocular lens implantation. *American Journal of Ophthalmology*.

[13] Pérez-Vives C., Ferrer-Blasco T., García-Lázaro S., Albarrán-Diego C., Montés-Micó R. (2014). Optical quality comparison between spherical and aspheric toric intraocular lenses. *European Journal of Ophthalmology*.

[14] Rajabi M. T., Korouji S., Farjadnia M. (2015). Higher order aberration comparison between two aspherical intraocular lenses: MC6125AS and Akreos advanced optics. *International Journal of Ophthalmology*.

[15] Johansson B., Sundelin S., Wikberg-Matsson A., Unsbo P., Behndig A. (2007). Visual and optical performance of the Akreos adapt advanced optics and Tecnis Z9000 intraocular lenses: Swedish multicenter study. *Journal of Cataract and Refractive Surgery*.

[16] Baghi A. R., Jafarinasab M. R., Ziaei H., Rahmani Z. (2008). Visual outcomes of two aspheric PCIOLs: Tecnis Z9000 versus Akreos AO. *Journal of Ophthalmic & Vision Research*.

[17] Nochez Y., Majzoub S., Pisella P. J. (2011). Effect of residual ocular spherical aberration on objective and subjective quality of vision in pseudophakic eyes. *Journal of Cataract and Refractive Surgery*.

[18] Shentu X., Tang X., Yao K. (2008). Spherical aberration, visual performance and pseudoaccommodation of eyes implanted with different aspheric intraocular lens. *Clinical & Experimental Ophthalmology*.

[19] Jia L. X., Li Z. H. (2014). Clinical study of customized aspherical intraocular lens implants. *International Journal of Ophthalmology*.

[20] McKelvie J., McArdle B., McGhee C. (2011). The influence of tilt, decentration, and pupil size on the higher-order aberration profile of aspheric intraocular lenses. *Ophthalmology*.

